# PTH Gene Polymorphism and Breast Cancer Risk in Kazakhstan

**DOI:** 10.5195/cajgh.2014.175

**Published:** 2014-12-12

**Authors:** Nurgul Sikhayeva, Zhannur Abilova, Ivan Shtefanov, Abai Makishev, Ainur Akilzhanova

**Affiliations:** 1 National Center for Biotechnology, Astana, Kazakhstan; 2 Center for Life Sciences, Astana, Kazakhstan; 3 Department of Oncology, Astana Medical University, Kazakhstan

**Keywords:** breast cancer, parathyroid hormone gene, polymorphism

## Abstract

**Introduction:**

Breast cancer is the most common type of cancer among women. In Kazakhstan, breast cancer holds first place among causes of women death caused by cancer in the 45–55 year age group. Many studies have shown that the risk of acquiring breast cancer may be related to the level of calcium in the blood serum. One of the important regulators of calcium metabolism in the body is the parathyroid hormone. Single nucleotide polymorphisms in the gene encoding the parathyroid hormone (PTH) are associated with breast cancer development risk, and may modify the associative interaction between the levels of calcium intake and breast cancer. Experimental studies have shown that PTH gene has a carcinogenic effect. At least three studies showed a weak positive correlation between the risk of acquiring breast cancer and primary hyperparathyroidism, a state with high levels of PTH and often high levels of calcium. The aim of this investigation was to evaluate potential association between PTH gene polymorphism and breast cancer risk among Kazakhstani women.

**Methods:**

Female breast cancer patients (n = 429) and matched control women (n = 373) were recruited into a case – control study,. Genomic DNA was extracted from peripheral venous blood of study participants using Wizard® Genomic *DNA Purification Kit* (Promega, USA). Detection of PTH gene polymorphism (rs1459015) was done by means of the TaqMan® SNP Genotyping Assay of real-time PCR. Statistical analysis was conducted using SPSS 19.0.

**Results:**

PTH gene alleles were in Hardy–Weinberg equilibrium (*p* > 0.05). Distribution was 59% CC, 35% CT, 6% TT in the group with breast cancer and 50% CC, 43% CT, 6% TT in the control group. Total difference (between the group with breast cancer and the control group) in allele frequencies for PTH polymorphism was not significant (*p* > 0.05). No association was found between rs1459015 TT and breast cancer risk (OR = 1.039; 95%, CI 0.740 – 1.297; *p* = 0.893).

**Conclusion:**

We found no association between PTHrs1459015 polymorphism and breast cancer in our present study. Further studies are required to confirm our results and clarify role of PTH gene genotypes on breast cancer risk.

